# First Record of *Clonostachys rosea* (Ascomycota: Hypocreales) Entomopathogenic Fungus in the Mango Hopper *Amritodus atkinsoni* (Hemiptera: Cicadellidae)

**DOI:** 10.3390/pathogens11121447

**Published:** 2022-11-30

**Authors:** Abhishek Kumar Tamta, Renu Pandey, Jiten R. Sharma, Rajnish Rai, Mritunjoy Barman, Deeksha M. G., Debasis Mitra, Pradeep Kumar Das Mohapatra, Rokayya Sami, Amina A. M. Al-Mushhin, Fadi Baakdah, Yasser S. Mostafa, Sulaiman A. Alrumman, Mahmoud Helal

**Affiliations:** 1 Department of Entomology, College of Agriculture, Govind Ballabh Pant University of Agriculture and Technology, Pantnagar 263145, India; 2 School of Life Sciences, Central University of Gujarat, Gandhinagar 382030, India; 3 Department of Agricultural Entomology, Bidhan Chandra Krishi Vishwavidyalaya, Mohanpur 741252, India; 4 Department of Entomology, G. D. Goenka University, Gurugram 122103, India; 5 Division of Entomology, ICAR-Indian Agricultural Research Institute, New Delhi 110012, India; 6 Department of Microbiology, Raiganj University, Raiganj 733134, India; 7 Department of Food Science and Nutrition, College of Sciences, Taif University, P.O. Box 11099, Taif 21944, Saudi Arabia; 8 Department of Biology, College of Science and Humanities in Al-Kharj, Prince Sattam Bin Abdulaziz University, Al-Kharj 11942, Saudi Arabia; 9 Department of Medical Laboratory Technology, Faculty of Applied Medical Sciences, King Abdulaziz University, Jeddah 21589, Saudi Arabia; 10 Special Infectious Agents Unit, King Fahd Medical Research Center, King Abdulaziz University, Jeddah 21589, Saudi Arabia; 11 Department of Biology, College of Science, King Khalid University, Abha 61413, Saudi Arabia; 12 Department of Mechanical Engineering, Faculty of Engineering, Taif University, P.O. Box 11099, Taif 21944, Saudi Arabia; 13 Production Engineering and Mechanical Design Department, Faculty of Engineering, Mansoura University, Mansoura P.O. Box 35516, Egypt

**Keywords:** biological control, novo-entomopathogenic fungus, *Clonostaychs rosea*, mango hopper, media optimization, entomology, etiology

## Abstract

Mango hopper (*Amritodus atkinsoni* Lethierry) causes devastations in the early vegetative stage of the mango crop. The classical management of mango hopper is with systemic insecticides but their overuse has caused environmental pollution. Here, we have evaluated the entomopathogenic role of *Clonostachys rosea* through bioassay and optimized media for its large-scale culturing. The current study reveals the potentiality of *C. rosea* as entomopathogenic on *A. atkinsoni*. Initially, morphological and molecular characterization was used to validate local isolates’ identity as *C. rosea*. Further, we have evaluated the entomopathogenic role of *C. rosea* through a bioassay, where the highest mean mortality in *A. atkinsoni* was observed at a treatment concentration of 3 × 10^8^ conidia/mL, with 96.67% mortality after 168 h of infection. This work also provides insight into the laboratory-based media standardization for *C. rosea*, resulting in oatmeal agar media and broth as the most suitable artificial media, and 20 °C temperature for its mass culture. Thus, *C. rosea* is a novo-entomopathogenic fungus on *A. atkinsoni* and has a high potency to be included in the management of mango hopper pests.

## 1. Introduction

*Mangifera indica* L. (Sapindales: Anacardiaceae) is a fruit crop that is cultivated in India, China, Thailand, Mexico, Pakistan, the Philippines, Indonesia, Brazil, Nigeria, and Egypt. India is the hub of mango diversity and is also one of the largest producers with an annual production of 18.0 million tons. Though India has the potential for higher production, insect pests act as a stumbling block to obtaining better growth and production of this crop. To date, 400 species of insect pests have been recorded to damage mango [1]. Among them, *Amritodus atkinsoni* Lethierry (Hemiptera: Cicadellidae) is serious insect pests of this crop [2]. Active nymphal and adult stages can affect the vigor of the trees by sucking the sap [3,4,5]. Several synthetic insecticides have been used to manage such a notorious insect pest [6]. However, continuous and heavy use of insecticides resulted in deleterious complications such as insect resistance, resurgence, and accumulation of soil and fruit residues [7]. These problems can thus be sustainably addressed by developing alternative methods such as biological control agents. In this perspective, screening of the potential entomopathogenic fungus *Clonostachys rosea* was carried out in this study. This fungus is present in various cultivated and non-cultivated areas, mostly in neutral to alkaline pH soils [8,9,10]. This myco-etiological agent has been classified as pathogenic to fungi, as an endophytic-antagonistic in plants, and as entomopathogenic [9,10,11]. However, only a few studies have characterized the entomopathogenic nature of this fungus. To date, *C. rosea* infection has been recorded on *Oncometopia tucumana* Schröder (Hemiptera: Cicadellidae), *Sonesimia grossa* Signoret (Hemiptera: Cicadellidae), *Anopheles stephensi* Liston (Diptera: Culicidae), *Culex quinquefasciatus* Say (Diptera: Culicidae), *Hypera postica* Gyllenhal (Coleoptera: Curculionidae), *Carpomyia vesuviana* Costa (Diptera: Tephritidae), *Bemisia tabaci* Gennadius (Hemiptera: Aleyrodidae), and *Aphis gossypii* Glover (Hemiptera: Aphididae) [10,12,13,14,15]. Additionally, *C. rosea* is a potential biocontrol agent for several insect pests, pathogenic fungi, and nematode parasites [16].

Although some investigations on the entomopathogenic nature of *C. rosea* have been documented, the nutrition and media optimization for large-scale culturing remain understudied [17,18,19]. The present study is also the first documentation of the entomopathogenic nature of *C. rosea* against the mango hopper. In addition, its efficacy as a biocontrol agent was assessed by performing an *in vitro* bioassay on the mango hopper. 

## 2. Materials and Methods

### 2.1. Collection, Isolation, and Culture Conditions of C. rosea

*A. atkinsoni* infected by *C. rosea* were collected from mango trees (*M. indica*) found at the College of Agriculture, G. B. Pant University of Agriculture and Technology, Pantnagar (29 °N and 79.3 °E) during the onset of winters and summers (2018–19). The samples were subjected to fungal isolation by following the Inglis et al. [20] procedure at the insect pathology laboratory, Department of Entomology, G. B. Pant University, India. Initially, the samples were soaked in distilled water for 24 h to activate the saprophytes or fungus of interest present on the surface of the cadaver. Subsequently, the cadavers were subjected to 0.3% sodium hypochlorite solution treatment to get rid of the externally grown fungi. The internally grown fungi were isolated on potato dextrose agar (PDA) media and the culture was maintained as a stock culture for further studies.

### 2.2. Morpho-Molecular Characterization of C. rosea

The identification of *C. rosea* was confirmed by morphological and molecular characterization. For the morphological study, scanning electron microscope (SEM)-based analysis was performed. For SEM analysis, the *C. rosea* hypha was mounted on glass slides, followed by drying and fixation using 2.5% glutaraldehyde for 2–3 h at 4 °C. The slides were washed with 0.1 M sodium phosphate buffer (7.4 pH, 3–5 min wash). The samples were dried using a KTech 850 critical point drier (Quorum Technologies, Lewes, UK), followed by a gold coating of the samples in a JFC 1600 gold coater (JEOL, Tokyo, Japan). The samples were finally subjected to analysis in a JSM 6610 SEM (JEOL, Tokyo, Japan). Parameters such as colony color, pigmentation of mycelium, conidiophore size, types of branches, phialide size, and size of conidia were all compared with the available literature [21,22,23,24,25,26]. Further, samples were sent to the Indian Type Culture Centre, IARI, Delhi, India, for validation of the identity of the local isolates on the basis of ‘phenotypic and physiological characters’. 

The morphological identification of the local isolates was corroborated with molecular identification based on the internal transcribed spacer (ITS) sequencing and phylogenetic analysis. ITS1 and ITS4 regions were chosen to validate the molecular identification in this study. Genomic DNA was isolated from the fungal cultures grown on PDA by the cetyl trimethyl ammonium bromide method [27] and further subjected to quality evaluation by 0.8% agarose gel electrophoresis. The PCR amplification of the ITS-rDNA region was performed using primers ITS1 and ITS4 (TCCGTAGGTGAACCTGCGG and TCCTCCGCTTATTGATATGC, respectively). The PCR was performed in a 25 μL reaction mixture using the following conditions: initial denaturation at 94 °C for 1 min followed by 35 cycles, each consisting of final denaturation at 94 °C for 30 sec, annealing temperature at 55 °C for 30 sec, initial extension for 1 min, and final extension at 72 °C for 10 min. PCR-amplified products were subjected to a 1.2% agarose gel run for confirmation. A 1kb DNA marker was used to estimate the proper band size of amplified products and they were visualized and photographed using a Gel Doc XR system (Bio-Rad Laboratories) [28]. 

The 18S rDNA region was sequenced to confirm the identity of the organism. The PCR product was sequenced in both directions by using the Sanger di-de-oxy method. A search for homologous sequences was performed by BLAST analysis at NCBI (http://ncbi.nlm.nih.gov/BLAST, accessed on 13 May 2019. Multiple alignments for homology search were performed using the Clustul W algorithm software. The MEGA7 software was used to construct dendrograms of the isolate from the current study and reference sequences, retrieved from GenBank [29,30].

### 2.3. Media Optimization for Mass Culturing of C. rosea

Fungi were cultured on PDA, Sabouraud’s dextrose agar + 1% yeast (SDAY), Sabouraud’s maltose agar + 1% yeast (SMAY), oatmeal agar (OMA), and Czapek–Dox agar (CZA) prepared as solid media following the standard protocol. Similarly, liquid media were prepared from the same constituent excluding agar-agar. One hundred milliliters of the sterile liquid media in conical flasks of 250 mLcapacity was prepared and used throughout the experimental procedures [26]. Inoculation of 0.7 cm bits of pure culture for solid and liquid media was carried out according to the study parameters such as mean radial growth, mean conidia per bit, and mean germination percentage of conidia in case of solid media and mean fresh weight or mean dry weight in case of liquid media. All these factors were further studied at 15, 20, 25, and 30 °C considering temperature as a prime and relevant factor for the vegetative and reproductive development of entomopathogenic fungi [31]. 

### 2.4. Preparation of C. rosea Spore Suspension for Pathogenicity Assays 

*Clonostachys rosea* was cultured on PDA for 15 days to develop spores on petri plates. With the help of a measuring cylinder, 10 mL of distilled water was poured onto the plates, and two drops of 0.01% Tween 80 were added. This was followed by scraping off mycelia from the plates into a suspension using a sterile infection loop. The suspension containing spores was filtered through a sterilized muslin cloth, serially diluted, and counted on a Neubauer counting chamber to achieve spore count. These spore suspensions were later used for the bioassay against *A. atkinsoni*.

### 2.5. Bioassay of C. rosea

To study the virulence of *C. rosea*, a bioassay on *A. atkinson* L. was carried out. A total of 30 insects (each treatment, 3 replications and each replication had 10 insects) with four concentrations of suspensions (1 × 10^4^, 1.5 × 10^5^, 2 × 10^6^, 2.5 × 10^7^, and 3 × 10^8^ spores per mL concentration) were assessed at consecutive 24, 48, 72, 96, 120, 144, 168, 192, 216, and 240 h time intervals, with control [10]. Finally, the per cent mortality was calculated by IBM-SPSS v16.0 for statistical probe and Duncan’s multiple range test (DMRT) at a 5% significance level. The etiology and epidemiology were studied for parameters such as the time required for adhesion, germination, colonization of fungus, first insect death, and last insect death.

## 3. Results

### 3.1. Collection, Isolation, and Culture Conditions of C. rosea

**Figure 1:**
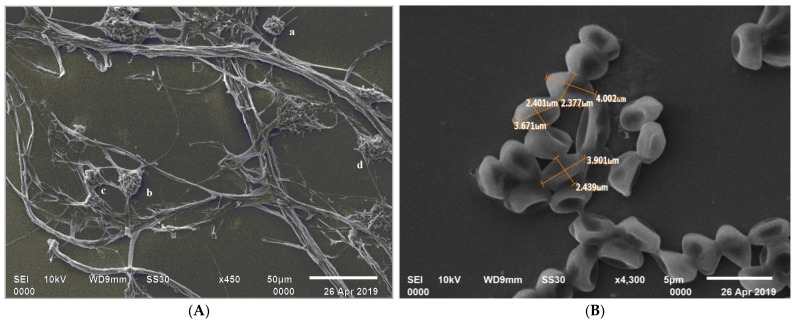
Mycelial growth and conidial development of *C. rosea*; (**A**) mycelial growth clustering and forming of spherical aggregates (a, b, c, and d); (**B**) SEM photograph showing conidial dimensions.

The isolate *C. rosea* 11031.19 was deposited at the Indian Type Culture Centre, IARI, Delhi, India. Further, the microscopic observation revealed a mycelium with branching hyphae and septate that were intertwined with each other. The mycelial growth led to the formation of round aggregates in various regions as observed with SEM (Figure 1A). The conidiophores appeared to have developed from the ropes of hyphae. The conidiophores were branched in verticillate (primary) and penicillate (secondary) forms. Phialides were flask-shaped with singular apices, unicellular, hyaline, oval, with curved-shaped conidia. The endings of the verticillate conidiophores showed a gelatinous matrix enveloping conidia and encompassing a mass of inclusion spherical aggregates. The Olympus CX33 microscope measurements revealed that conidiophores were 83.2–95.8 μm with an angle of 12.5°–154.4°. The size of phialides (40× objective lens) was 86.7–111.4 μm with an angle for alignment in the range of 24.9°–80.7°. The dimensions of mycelium knots were 22.0–24.9 µm. The conidial dimensions observed on SEM were in the range of 2.2–2.3 μm × 4.0–4.3 μm (Figure 1B) and the hyphae had a width of 2.0–2.3 μm. 

### 3.2. Molecular Identification of Local Isolates

**Figure 2:**
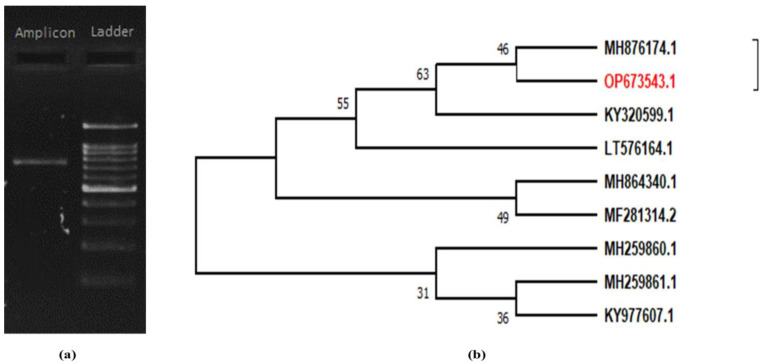
Molecular identification of *C. rosea* by (**a**) ITS PCR amplicon and (**b**) NJ phylogenetic tree.

The rDNA sequence of the ITS region was estimated to be ~700 bp, confirmed with ladder specification (Figure 2a,b). Analysis with NCBI BLAST resulted in a 100% identity with *C. rosea.* The sequence was assigned with accession number OP673543.1. Two clades were formed on the phenogram using the consensus ITS sequence from the local isolates and ten accessions were obtained from NCBI based on a maximum identity score. The local isolate ITS sequence fell in a subclade with maximum similarity with *C. rosea* isolated from Korea (Figure 2b).

### 3.3. Media Optimization for Mass Culturing 

The studies of solid media at 20 °C for mean growth (Table 1) showed the highest mean radial growth in OMA, followed by SMAY, CZA, and SDAY with the lowest mean radial growth obtained in PDA. When mean spores/bit were studied at the same temperature, it was found that the most spores were obtained from OMA, followed by SDAY, CZA, and SMAY and the least production was obtained from PDA. We also evaluated mean germination percentage and found that the highest germination was obtained from OMA, followed by PDA, SDAY, and CZA with the lowest frequency seen in SMAY. After analyzing the mean radial growth, mean conidia/bit, and mean germination, the best solid media for *C. rosea* was OMA, followed by SDAY, CZA, and SMAY. PDA at 20 °C was found to be the least effective media in contrast with the data for the other temperatures used and displayed in Table 1.

Similarly, at 20 °C, the highest mean fresh weight biomass was achieved (Table 2) in OMB followed by SMYB, PDB, and SDYB. The lowest mean fresh biomass was obtained from CZB. The data for dry biomass were also recorded showing the highest mean dry biomass (mycelium + conidia) obtained from OMB followed by SMYB, SDYB, and PDB. The lowest mean dry biomass was achieved in CZB. The results from the studies carried out at 15, 25, and 30 °C are presented in Table 2.

### 3.4. Efficacy of C. rosea against A. atkinsoni

**Figure 3:**
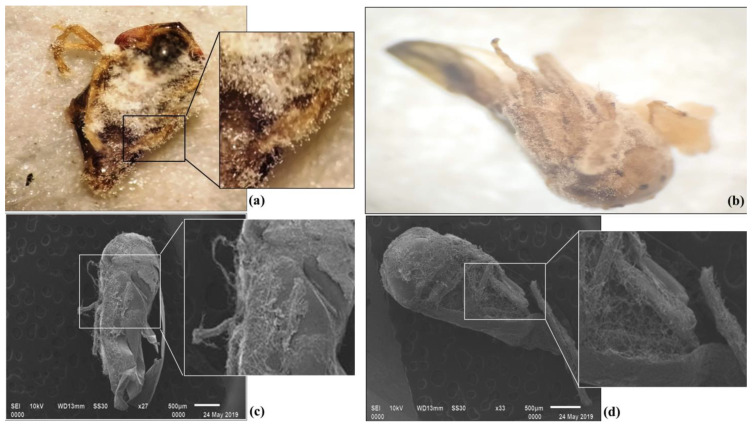
Specimens of *A. atkinsoni* were parasitized by *C. rosea* five days after infection assessed by bright field microscopy (**a**,**b**) and SEM (**c**,**d**).

The response for pathogenicity against mango hopper (Figure 3a–d) was high in two concentrations (3 × 10^8^ and 2.5 × 10^7^), with the mortality response (13.33 and 3.33%) augmenting on the 3rd day after exposure. However, lower concentrations required a longer exposure period for mortality to be achieved. At 2 × 10^6^ conidia/mL and 1.5 × 10^5^ conidia/mL concentrations, the mortality response (3.33 and 3.33%) started on the 4th day. For the lowest concentration of 1 × 10^4^ conidia/mL, the mortality response (3.33%) started on the 5th day after exposure. On the 7th day after exposure, the mean mortality was highest at 3 × 10^8^ spores/mL concentration (96.67%), followed by 2.5 × 10^7^ spores/mL concentration (70.00%), and 2 × 10^6^ spores/mL concentration (53.33%). However, in other lower concentrations, mortality was less than 50% (Table 3). The etiology and epidemiology of *C. rosea* on mango hoppers were observed by considering parameters such as the time required for adhesion, germination, colonization of fungi, first insect death, and last insect death, which were 24, 36, 48, and 192 h, respectively. Out of 30 mango hoppers, 29 were dead within 168 h. Therefore, the obtained data suggest that a colonization period of 48 h was required, whereas for complete parasitization, it would take 120–132 h.

## 4. Discussion

*Clonostachys rosea* is a mitosporic-hypocreale fungus with a biotrophic nature and also a potential biological control agent against various pathogens and pests. There are reports of its isolation from various crops (barley, strawberry, rose, lettuce, cocoa, etc.), insects, nematodes, and even wine [12,13,14,15,32,33,34,35]. In the present study, *C. rosea* was found to be a parasite of a major insect pest of mango, *A. atkinsoni* [2]. There are very few available studies documenting the entomopathogenic nature of this fungus and, to the best of our knowledge, a study on mango hopper was never conducted. Meanwhile, this fungus has a role in controlling grey mold, crown rot, and others as a potential biocontrol agent, thus it can be considered for pest management programs [25]. The majority of studies regarding *C. rosea* biology, media standardization, mass culture, and application for disease management, biotransformation, biodegradation, and biological energy sources have been performed considering only plant pathology applications [16,24,36]. Although, to date, insects such as *O. tucumana*, *S. grossa*, *H. postica*, *C. vesuviana*, *B. tabaci*, *Galleria mellonella*, and *Aphis gossypii* have been studied with *C. rosea*, the presence of this fungus in the microhabitat of a highly remunerative crop such as mango has not been reported yet [10,12,13,14,15,37]. 

Our data showed susceptibility of *A. atkinsoni* to various conidial concentrations of *C. rosea* (Table 3). The mortality response in the case of the highest concentration (3 × 10^8^ conidia/mL) was observed on the 3rd day onwards after the exposure, wherein, the mortality recorded was found to be 13.3%. On increasing the exposure up to 7 days, 96.6% mortality was observed. This mortality response is consistent with the study conducted by Prabhu and Kumar [12], where a concentration of 10.52 × 10^6^ spores/mL caused mortality of *A. stephensi* (92.8%) and *C. quinquefasicatus* (71.9%) in 72 h. Similarly, Mustafa et al. [13] from the northern region of Iraq reported 55.1% mortality of alfalfa weevil (*Hypera postica*) by *C. rosea* after 6 days of inoculation. Additionally, Mahmoudi et al. [14] demonstrated that a conidial suspension of 10^10^ spores/mL induced mortality of 46% when assessed after 7 days of inoculation. Lethal time (LT50) and LC50 obtained against *C. vesuviana* were 4.6 days and 5.1 × 10^4^ spores/mL, respectively. The study by Anwar et al. [15] showed that 4 × 10^8^ spores/mL resulted in a 16.33% mortality in *B. tabaci* nymphs after 4 days and 50.42% after 6 days of *C. rosea* infection. Thus, *C. rosea* appears as an effective biocontrol agent with a wide host range, including *A. atkinsoni*.

The media optimization for *C. rosea* mass culturing suggests that OMA was the best in comparison with the other four media. This study identified and specified OMA solid media at 20 °C for mass culturing. However, for liquid media optimization, OMB showed the highest biomass. The best biomass weight could be achieved from 20 °C to 25 °C. These results are consistent with the findings of Isaac [24], where oatmeal extract was shown to produce a flourishing woolly growth of *C. rosea* and the optimum temperature for growth was recorded at 25 °C. In addition, several other studies depicted OMA as the best choice of media [8,9,38]. Furthermore, in the present study, more emphasis was given to the temperature-dependent development of *C. rosea* while optimizing media, as, for any biological control agent, temperature-dependent development aspects are the most significant environmental factor. Therefore, our approach further corroborated the observation by Perdikis and Lykouressis, and Edelstein et al. [30,39]. Thus, in the present study, OMA was used to mass-produce *C. rosea* which was utilized for bioassays. Since nutrition is an important factor for the production of effective conidia and to achieve strong virulence of entomopathogenic fungi, optimization of the culture media appears as an important step when a new host–pathogen interaction is recorded, as carried out in the present study [34,40]. Moreover, media optimization is of prime importance since it aids in the large-scale production of metabolites and fungal colony components of the desired fungi. Therefore, considering the importance of suitable nutritional media to multiply *C. rosea*, we designed the media and optimized the conditions required for mass production of *C. rosea* to obtain high-quality spores and develop a suitable formulation.

## 5. Conclusions

The present study showed that *C. rosea* is a potential agent for infecting and killing *A. atkinsoni* in the mango ecosystem. Moreover, the optimization of media for studying any new host–pathogen interaction is essential for obtaining effective conidia to be used in a biocontrol strategy. Deeper knowledge on the pathogenicity of entomopathogenic *C. rosea* will help us in managing field pest problems. These observations will further aid in developing better strategies for biointensive insect pest management. The present investigation therefore has a potential future prospect to be extrapolated, as it can be inferred that *C. rosea* is a new entomopathogenic fungus against mango hoppers with a potential to manage their population.

## Figures and Tables

**Table 1:** Studies of solid media at 15, 20, 25, and 30 °C for mean radial growth (cm), mean conidia/bit, and mean germination.

	15 °C			20 °C			25 °C			30 °C		
Media	Radial Growth (cm)	Conidia/Bit	Germination (% )	Radial Growth (cm)	Conidia/Bit	Germination (% )	Radial Growth (cm)	Conidia/Bit	Germination (% )	Radial Growth (cm)	Conidia/Bit	Germination (% )
		−10^6^			(10^6^)			(10^6^)			(10^6^)	
SDAY	2.67	2.37	78.52	5.84	4.71	76.64	5.29	0.5	82.64	3.18	0.73	78.6
SMAY	3.55	0.37	76.16	7.53	3.65	74.32	7.2	0.31	78.84	2.66	0.45	80.45
PDA	1.7	0.27	75.34	3.83	1.91	79.16	5.66	1.63	74.67	1.4	0.53	80.38
OMA	4.65	2.79	86.77	8.21	9.81	92.25	7.74	4.4	86.94	4.12	1.73	83.66
CZA	2.41	2.35	72.79	6.56	3.83	75.67	6.59	0.85	74.86	2.79	0.13	74.81
SE	0.54	0.37	1.32	0.56	0.53	1.21	0.74	0.53	1.61	0.56	0.18	1.19
CD at 5%	0.16	0.11	3.983	0.17	0.16	3.68	0.22	0.16	4.86	0.17	0.53	3.58
CV	3.58	4.57	3.39	1.74	2.23	3.05	2.29	2.23	4.04	3.94	4.96	2.99

**Table 2:** Mean fresh and dry biomass weights (g) at 15, 20, 25, and 30 °C in response to different liquid media.

	15 °C		20 °C		25 °C		30 °C	
Media	Fresh	Dry	Fresh	Dry	Fresh	Dry	Fresh	Dry
SMAY	5.55	0.87	5.58	1.04	7.37	1.13	0.85	0.69
SDYB	5.39	0.9	4.67	1.02	7.66	1.03	3.17	0.88
PDB	4.36	0.8	5.22	0.83	4.76	0.73	1.65	0.75
OMB	7.56	1.07	8.87	1.28	7.95	1.39	4.66	0.96
CZB	2.13	0.64	2.26	0.75	6.56	0.28	4.41	0.77
SE	0.49	0.23	0.6	0.26	0.56	0.12	0.49	0.12
CD at 5%	0.15	0.74	0.19	0.81	0.18	0.4	0.15	0.4
CV	1.7	4.8	1.96	4.54	1.41	2.43	2.87	2.73

**Table 3:** Mean mortality per cent for different concentrations against *A. atkinsoni*.

Concentration (Conidia/mL)	Day 1	Day 2	Day 3	Day 4	Day 5	Day 6	Day 7
T1 (3 × 10^8^)	0.0 ± 0.0 ^a^	0.0 ± 0.0 ^a^	13.3 ± 5.7 ^b^	33.3 ± 15.2 ^b^	56.6 ± 11.5 ^c^	76.6 ± 5.7 ^d^	96.6 ± 5.7 ^e^
T2 (2.5 × 10^7^)	0.0 ± 0.0 ^a^	0.0 ± 0.0 ^a^	3.3 ± 5.7 ^a^	13.3 ± 15.2 ^a^	33.3 ± 20.8 ^b^	46.6 ± 15.2 ^c^	70.0 ± 10.0 ^d^
T3 (2 × 10^6^)	0.0 ± 0.0 ^a^	0.0 ± 0.0 ^a^	0.0 ± 0.0 ^a^	3.3 ± 5.7 ^a^	13.3 ± 15.2 ^ab^	33.3 ± 15.2 ^bc^	53.3 ± 5.7 ^c^
T4 (1.5 × 10^5^)	0.0 ± 0.0 ^a^	0.0 ± 0.0 ^a^	0.0 ± 0.0 ^a^	3.3 ± 5.7 ^a^	13.3 ± 11.5 ^ab^	20.0 ± 17.3 ^ab^	16.6 ± 11.5 ^b^
T5 (1 × 10^4^)	0.0 ± 0.0 ^a^	0.0 ± 0.0 ^a^	0.0 ± 0.0 ^a^	0.0 ± 0.0 ^a^	3.3 ± 5.7 ^a^	6.6 ± 5.7 ^a^	6.6 ± 5.7 ^ab^
Control	0.0 ± 0.0 ^a^	0.0 ± 0.0 ^a^	0.0 ± 0.0 ^a^	0.0 ± 0.0 ^a^	0.0 ± 0.0 ^a^	0.0 ± 0.0 ^a^	0.0 ± 0.0 ^a^

Treatment response, mean values (±SE) following the same superscript letters (a, b, c, d, and e) are significantly different from each other; mean values (±SE) that are not followed by the same superscript letters (ab and bc) do not differ significantly from one another (*p* = 0.05%).

## Data Availability

Data is contained within the article.
